# Colonic Medium-Chain Fatty Acids Act as a Source of Energy and for Colon Maintenance but Are Not Utilized to Acylate Ghrelin

**DOI:** 10.3390/nu13113807

**Published:** 2021-10-26

**Authors:** András Gregor, Sandra Auernigg-Haselmaier, Slave Trajanoski, Jürgen König, Kalina Duszka

**Affiliations:** 1 Department of Nutritional Sciences, University of Vienna, Althanstrasse 14, 1090 Vienna, Austria; andras.gregor@univie.ac.at (A.G.); sandra.haselmaier@univie.ac.at (S.A.-H.); juergen.koenig@univie.ac.at (J.K.); 2 Core Facility Computational Bioanalytics, Medical University of Graz, 8010 Graz, Austria; slave.trajanoski@medunigraz.at

**Keywords:** medium-chain fatty acids, gastrointestinal tract, microbiota, nutrition

## Abstract

The capacity of microbiota to produce medium-chain fatty acids (MCFA) and related consequences for the gastrointestinal (GI) tract have never been reported before. We verified the impact of nutrition-related factors on fatty acid (FAs) production and found that caloric restriction decreased levels of most of MCFAs in the mouse cecum, whereas overnight fasting reduced the levels of acetate and butyrate but increased propionate and laurate. A diet high in soluble fibre boosted the production of short-chain fatty acids (SCFA) and caproate whereas a high-cellulose diet did not have an effect or decreased the levels of some of the FAs. Rectal infusion of caprylate resulted in its rapid metabolism for energy production. Repeated 10-day MCFA infusion impacted epididymal white adipose tissue (eWAT) weight and lipid accumulation. Repeated infusion of caprylate rectally tended to increase the concentration of active ghrelin in mice plasma; however, this increase was not statistically significant. In Caco-2 cells, caprylate increased the expression of Fabp2, Pdk4, Tlr3, and Gpr40 genes as well as counteracted TNFα-triggered downregulation of Pparγ, Occludin, and Zonulin mRNA expression. In conclusion, we show that colonic MCFAs can be rapidly utilized as a source of energy or stored as a lipid supply. Further, locally produced caprylate may impact metabolism and inflammatory parameters in the colon.

## 1. Introduction

Recent years brought great interest in microbiota followed by the discovery of multiple interactions between host and gut bacteria. Short-chain fatty acids (SCFA), products of colonic bacteria fermentation, came into focus as the main factor of beneficial interaction between the gut and its inhabitants being substantial for colon health, metabolism, and inflammatory regulation [1,2,3,4,5]. However, other kinds of fatty acids (for example, medium-chain fatty acids (MCFA)) have not been associated with gut bacteria so far. Certain yeast strains were reported to produce one of the MCFAs, caprylate (C8) [6]. For industrial purposes, MCFAs can be generated by *Clostridium kluyveri* [7,8,9], a bacterial strain, which resides in the intestine of rumen [10,11]. However, reports concerning gut bacteria-derived MCFAs in non-rumen animals are lacking. Interestingly, MCFAs are known for their anti-bacterial and anti-fungal properties towards selected bacterial strains [12,13]. On average, the intake of MCFA through the modern human diet is less than 2% of dietary energy [14]. MCFAs are taken up in the intestine and metabolized in the liver faster than LCFAs [15,16,17] as a consequence of passive diffusion to the portal vein and rapid transport to the liver of MCFAs versus relatively slow absorption of long-chain fatty acids (LCFA) into the lymphatic system. When compared to LCFAs, supplementing MCFAs has been proven to stimulate weight loss and increase energy expenditure [18,19,20,21,22,23]. MCFAs activate the transmembrane receptor G-protein-coupled receptor 40 (GPR40) [24] which has a particularly high expression in the insulin-producing islet cells of the pancreas but also in the central nervous system, adipose tissue, taste buds, liver, stomach, and gut [25,26,27]. GPR40 is involved in the regulation of blood insulin [28] and in the secretion of incretins (GLP-1, GIP) from endocrine cells of the gastrointestinal (GI) tract [27,28].

Caprylate (C8) naturally occurs in the human diet in small amounts in coconut oil, palm kernel oil, and milk products [14,29]. Importantly, caprylate binds to and thus activates the hunger-signalling hormone ghrelin [30]. Ghrelin modulates dopamine levels and neuron activity in the brain, prompting food-seeking behaviours and the anticipation of a meal. Likewise, it mediates food-related reward, enhances taste responsiveness, and increases the appeal of high-calorie choices [31,32,33,34,35,36,37], but also stimulates other self-indulgent activities [38]. Ghrelin levels increase rhythmically preceding the regular mealtime [39,40,41,42,43] and thus enhance pre-prandial gastric motility and gastric acid secretion [44,45]. The levels also rise during fasting and are reduced by refeeding [46]. While obesity is accompanied by decreased levels of circulating ghrelin to signal nutrient abundance [47,48], obesity triggered by a high-fat diet in mice is characterised by ghrelin resistance [49] further contributing to dysregulation of appetite. Caprylate influences hunger perception (and potentially body weight) by activating ghrelin. However, infusion or oral delivery of caprylate in different studies resulted in contradictory outcomes on subsequent food intake [50,51] and activation of ghrelin [52,53]. Therefore, the source of caprylate used for ghrelin acylation remains controversial. Up to now, intrinsic, microbiota-derived caprylate has not been investigated as a ghrelin activator. Besides the potential role in ghrelin acylation, microbiota-derived caprylate may play a role locally in the distal gastrointestinal (GI) tract, as MCFAs can protect from induction of colitis and display anti-inflammatory properties in the colon [54,55,56,57,58]. Accordingly, Inflammatory Bowel Diseases (IBD) patients show decreased fecal levels of MCFAs, including caproate (C6), heptanoate (C7), and caprylate [59]. Thus, similarly to SCFAs, MCFAs have a great (but unexplored) potential as mediators of gut health and for the interaction between the host and enteric microbiota. We hypothesized that endogenously produced caprylate serves to acylate ghrelin and has a local impact on the colon.

## 2. Materials and Methods

### 2.1. Mice Experiments

Male C57BL/6NRj mice were purchased from Janvier Inc. Labs (Le Genest, France) and housed in standard specific-pathogen-free (SPF) conditions with wooden (Lignocel select) bedding. Mice from control (ad libitum), caloric restriction (CR), and overnight (ON) fast groups were fed a standard chow (V1535 R/M-H Extrudate; Ssniff Spezialdiäten GmbH, Soest, Germany).

For the CR and ON fast experiment, the animals were randomly divided into experimental and control ad libitum groups. The CR mice were submitted to two weeks CR with 75% of normal food intake as reported previously [60,61]. For the ON fasting experiment, food was removed for 16 h with free water access. 

In order to compare the impact of fibre on SCFA and MCFA production, another group of mice was fed with a control diet containing 5% of mixed fibres, or with a high-fibre diet containing either 20% soluble fibre (oligofructose and pectin) or 20% insoluble fibre (cellulose) (all custom made by SSNIFF Spezialdiäten GmbH). The detailed diet composition is presented in the Supplementary Materials file.

For the infusion experiments, one group of ON fasted mice received 50 μL saline, 50 μL 600 μM of sodium caproate, sodium caprylate, or sodium laurate (Sigma-Aldrich) either by infusion in the colon or by gavage using a 3.5 cm-long elastic blunted gavage needle. For the rectal infusion, the needle was left in the colon for approximately 1 min and slowly withdrawn to avoid leaking the solution out. To assess hunger, a food pellet was placed 10 min after the infusion in the cage and the time it took the mice to begin consumption was measured with a stopwatch. 

Another group of mice received a daily rectal infusion treatment of the same saline control (50 μL) or 50 μL 200 μM of FA solutions (sodium caproate, sodium caprylate, or sodium laurate) for 10 days. To measure food intake, the weight of all food pellets was measured at the indicated time points over 24 h or every day over a 10 day infusion period.

For measurement of ghrelin concentration, 6 h fasted mice were infused with 50 μL of 200 μM sodium caprylate or sodium laurate, and blood samples were collected from the tail vein before (0 h) as well as 15 min and 30 min after the infusion.

For the estimation of caprylate metabolism, 50 μL of ^13^C labelled 200 μM sodium caprylate solution (Sigma-Aldrich, St. Louis, MI, USA) were infused into the colon. Next, each mouse was placed in a separate sealed jar with a rubber septa fixed on the top. A sample of 5 mL of air from the jar was collected using a syringe and a needle, and the syringe was closed using a stop cock before removing the needle from the jar. The collected air was released to a 10 mL glass vial with rubber septa. The samples were collected 30 s (0 min time point), 15 min, 30 min, and 1 h after placing the mouse in the jar.

For the estimation of colonic caprylate distribution in peripheral tissues, 50 μL of ^13^C labelled 200 μM sodium caprylate solution (Sigma-Aldrich) were infused into the colon, and the tissues were dissected 1 h later.

On the day of sacrifice, food from the cages of control ad libitum-fed mice was removed 2 h before the dissection. All animals were euthanized via isoflurane overdose, with blood drawn by cardiac puncture. Organ weights (including the stomach and cecum with their content as well as epididymal adipose tissue (eWAT) and liver) were recorded. EDTA (10 μL/mL), aprotinin (20 μL/mL), and dipeptidyl peptidase (DPP) IV (10 μL/mL) were added to freshly collected blood prior to centrifugation (10 min at 3600× *g* 4 °C). Colonic mucosa scrapings, cecum content, eWAT, and liver were snap-frozen and stored at −80 °C until use. 

Plasma total and active ghrelin were measured using ELISA (Millipore, Merck, Vienna, Austria) following the manufacturer’s indication. Liver and eWAT triglycerides were quantified using the Triglyceride Colorimetric Assay Kit (Cayman Chemicals, Ann Arbor, MI, USA).

All animal experimentation protocols were approved by the Federal Ministry of Science, Research and Economy, Unit for Animal Experiments and Genetic Engineering in Austria (BMWFW-66.006/0017-WF/V/3b/2016). The experiments were performed in agreement with the Austrian Federal Act on Animal Welfare.

### 2.2. Cell Culture

Caco-2 cells were grown in high glucose DMEM supplemented with 10% foetal bovine serum, 100 U/mL penicillin, and 100 U/mL streptomycin (all from Sigma-Aldrich) under a humidified atmosphere of 5% CO_2_ at 37 °C. For the proliferation assay, cells were incubated with caprylate at a concentration range of 0.1 μM–0 mM for 4 h. Cell proliferation was measured using a BrdU assay (BioVision, Milpitas, CA, USA) according to the manufacturer’s protocol. For the other assays, Caco-2 cells were cultured for 20 days after reaching confluence. TNFα (Sigma-Aldrich) and caprylate were added to the culture to final concentrations of 10 ng/mL and 1 mM, respectively. Following 2 h incubation, the cells were collected in the lysis buffer of the RNeasy mini kit (Qiagen, Hilden, Germany).

### 2.3. SCFA and MCFA Detection

As reported previously [60], the detection of SCFA and MCFA was carried out with a derivatization method using LCMS. In brief, frozen cecum tissue samples were homogenized in Precellys homogenizing tubes. The extraction buffer contained methanol, chloroform, and water with a ratio of 2.5:1:0.5. After vortexing and centrifuging the samples for 5 min at 10,000 rpm at 4 °C, 600 μL of the supernatant were transferred to a new Eppendorf tube. The extraction was repeated with 400 μL extraction buffer and the supernatant was combined with the supernatant from the first extraction. 100 μL of the combined supernatant were dried in a SpeedVac concentrator for 60 min at 45 °C. Dried pellets were resuspended in 150 μL acetonitrile:water (1:1) and 40 μL 40 mM 2-NPH, 40 μL 250 mM EDC and 40 μL 3% pyridine. Samples were vortexed and incubated for 30 min at 60 °C. After cooling down at room temperature, samples were centrifuged at 14,000 rpm at 4 °C and dried in the SpeedVac concentrator for 75 min at 45 °C. To avoid phase separation, samples were resuspended in 50 μL acetonitrile:water (1:1) and diluted with 50 μL of acetonitrile:water (9:1). Samples were vortexed thoroughly and were transferred into HPLC vials. During the analysis, samples (10 μL) were kept at 10 °C and analysed via liquid chromatography coupled to mass spectrometry (LC-MS) using an Ultimate 3000 (Thermo Fischer Scientific, Waltham, MA, USA) and a micrOTOF-Q II (Bruker Daltonics, Bremen, Germany) with an Atlantis T3 3 μm column (2.1 × 150 mm, Waters, Milford, MA, USA). 

The column was kept at 40 °C. The ratio of mobile phase B (acetonitrile + 0.1% formic acid) was increased from 5% (0–2.5 min) to 90% (8 min), followed by a 5 min hold at 90%, then the column was washed with 95% mobile phase A (H_2_O + 0.1% formic acid) for 2 min. The retention time of acetate, propionate, butyrate, valerate, caproate, caprylate, caprate, and laurate was 8.0 min, 8.6 min, 9.0 min, 9.5 min, 10.0 min, 10.9 min, 12.3 min, and 14.9 min, respectively. In ESI negative mode the precursor ions of the adducts of acetate, propionate, butyrate, valerate, caproate, caprylate, caprate, and laurate were 194.05 m/z, 208.07 m/z, 222.08 m/z, 236.10 m/z, 250.12 m/z, 278.15 m/z, 306.18 m/z, and 334.21 m/z, respectively. LCMS grade solvents and chemicals were purchased from VWR Chemical (Fontenay-sous-Bois, France) or Sigma-Aldrich (Steinheim, Germany).

### 2.4. Gene Expression

RNA was isolated from colon mucosa and liver using the RNeasy mini kit (Qiagen, Hilden, Germany). Samples were thawed in lysis buffer and disrupted using a syringe and needle or the Precellys 24 homogenizer for mucosa and liver, respectively. RNA was extracted following the manufacturer’s recommendations. qScript cDNA synthesis kit SuperScript II Reverse Transcriptase (Quanta Biosciences, Gaithersburg, MA, USA) was used for the reverse transcription. Quantitative real-time PCR (qRT-PCR) reactions were carried out using the QuantaStudio 6 Flex (Applied Biosciences, CA, USA) with the PerfeCTa SYBR Green PCR Master Mix (Quanta Biosciences). The Supplementary Materials file lists the used primers.

### 2.5. Histology

Pieces of liver and eWAT were fixed in 4% buffered paraformaldehyde for 48 h at 4 °C followed by rinsing, dehydration, and embedding in paraffin. The paraffin blocks were cut into 4 μm sections and processed for hematoxylin and eosin (HE) staining. Digital images of the stainings were captured using Aiocam ERc 5S (Carl Zeiss Microscopy Ltd., Germany) microscope and Zeiss Efficient Navigation (ZEN) software. Adipocytes’ area was quantified using ImageJ 1.38e (https://imagej.nih.gov/ij/index.html, accessed on 21 June 2021).

### 2.6. Sequencing the 16S rDNA Genes and Metataxonomic Analysis

The cecal content samples were processed as previously published [60] for sequencing. In brief, the samples were homogenized following the protocol of the MagNA Pure LC DNA Isolation Kit III (Bacteria, Fungi) in a MagNA Lyser Instrument (all from Roche, Mannheim, Germany). The homogenized samples were incubated with lysozyme, followed by adding Proteinase K and incubation at 65 °C ON. Next, 250 μL lysed supernatant were used for DNA extraction using MagNA Pure LC DNA Isolation Kit III (Bacteria, Fungi) (Roche). PCRs reactions were run applying the FastStart High Fidelity PCR system. The reaction mix contained 5 μL of total DNA, 1x Fast Start High Fidelity Buffer, and 1.25 U High Fidelity Enzyme. The V1-V2 primers (27F—AGAGTTTGATCCTGGCTCAG and 375R—CTGCTGCCTYCCGTA) were applied with Illumina adapters for an indexing PCR reaction. The PCR reaction triplicates were pooled, normalized on a SequalPrep Normalization Plate (LifeTechnologies, Germany), and applied as a template in an indexing PCR reaction. The final sequencing library was purified using a 1% agarose gel and the QIAquick gel extraction kit (Qiagen, Germany), quantified with QuantiFluor ONE dsDNA Dye on a Quantus Fluorometer (Promega, Germany), and its quality was verified using an Agilent BioAnalyzer 2100 (Waldbronn, Germany). The 6 pM library was sequenced on a MiSeq desktop sequencer (Illumina, Netherlands) containing 20% PhiX control DNA (Illumina). The Galaxy web-based platform [62] and the QIIME2 2018.4 microbiome analysis pipeline were used to analyse FastQ raw reads. The data were pre-processed with DADA2 [63]. The feature representative sequences were classified with the QIIME2 against SILVA 16S rRNA database version 132 at 99% identity [64]. The feature abundance table was used for all subsequent analyses. Representative sequences were aligned with MAFFT de novo multiple sequence alignment [65] followed by the creation of a phylogenetic tree with FastTree [66]. The full data set with the sequencing results are available from our previous publication [60].

### 2.7. Statistics

For the comparison of the results of more than two groups, one-way ANOVA with Bonferroni post-hoc corrections for multiple testing was applied. Differences between two experimental groups were assessed using Student’s *t*-test with a statistical significance threshold set at *p* < 0.05. The statistical software COVAIN [67] under MATLAB environment was used for data transformation, alignment, and integrative analysis including correlation coefficients, hierarchical clustering, and one-way ANOVA. The bi-clustering was based on average linkage analysis of Euclidean distance between groups, calculated from Pearson’s rank correlation coefficient. The amount of each FA and bacteria OTUs were z-scored across all samples.

## 3. Results

**Figure 1:**
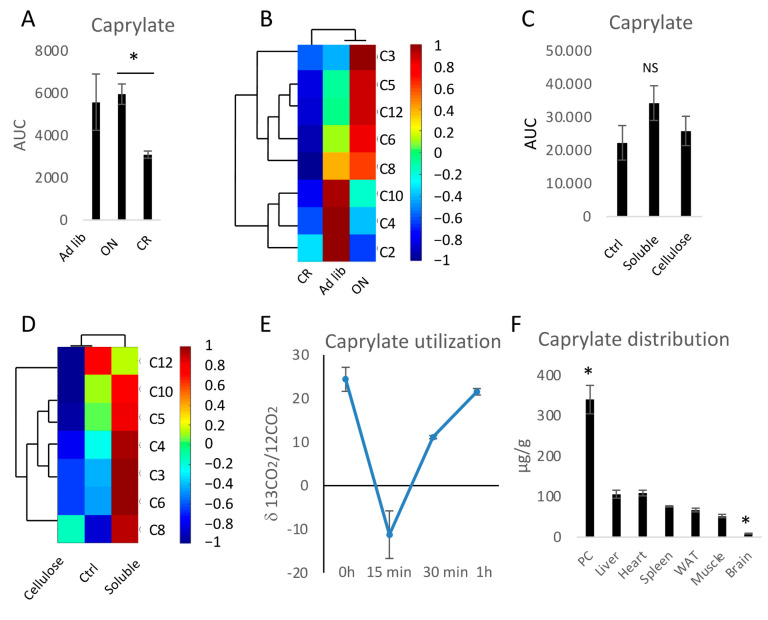
Medium-chain fatty acids (MCFA) found in mice cecum can be metabolized and exported to other organs. The levels of caprylate (**A**), other short-chain fatty acids (SCFA), and MCFA were measured in the cecum content of mice submitted to overnight fasting (ON) and caloric restriction (CR) (**B**). The levels of caprylate (**C**), SCFA, and MCFA (**D**) were compared between the cecum content of mice fed control (Ctrl), high soluble fibre, or high-cellulose diet. The ratio of ^13^C-labelled CO_2_ to ^12^C CO_2_ was measured in the breath air of mice rectally infused with ^13^C-labelled caprylate (**E**). The levels of ^13^C-labelled caprylate were measured in the proximal colon (PC), heart, liver, WAT, spleen, skeletal muscle, and the brain 1 h after rectal infusion of ^13^C-labelled caprylate (**F**). Statistical significance between the experimental groups in panels (**A**,**C**) was evaluated using two-tailed Student’s *t*-tests; *n* = 8; * *p* < 0.05. In panel (**F**), * indicates *p* < 0.05 versus all other tissues. NS stands for not statistically significant. Data are presented as mean ± SEM. Hierarchical clustering and heatmap analysis of FAs were based on Pearson’s correlation coefficient, which is depicted as colours showing positive correlation (red) or negative correlation (blue).

**Figure 2:**
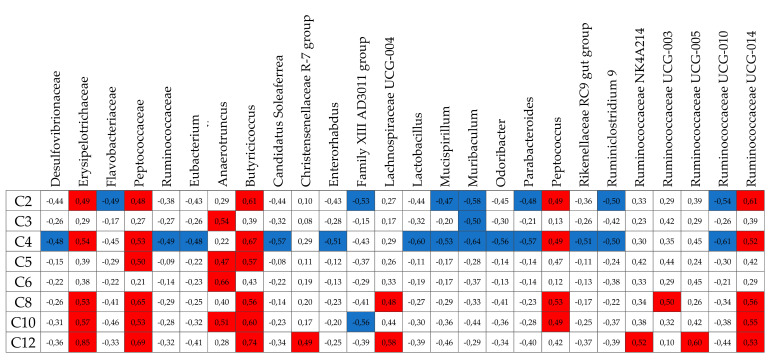
The occurrence of SCFA and MCFA correlates with fecal bacteria abundance. Correlation coefficients of FAs and bacteria are indicated in red colour for statistically significant positive correlations and in blue colour for statistically significant negative correlations; *p* < 0.05.

In a search for alternative, non-dietary sources of MCFAs that could serve to acylate ghrelin, we aimed to verify the capacity for endogenous caprylate production in the gut. Since dietary restriction results both in changes of ghrelin levels [46] and of microbiota composition [61], the levels of SCFA and MCFA were measured in the cecum of ad libitum fed, ON fasted and CR animals (Figure 1A,B and Figure S1A–G). ON fasting did not influence caprylate levels but CR decreased them (Figure 1A). Therefore, caprylate is present in the cecum; however, its level is not increased during fasting or CR when the levels of ghrelin are elevated. ON fasting increased levels of propionate, and laurate, while it decreased levels of acetate and butyrate showing a stronger impact on SCFA compared to MCFA (Figure 1B and Figure S1A–G). CR decreased levels of acetate and butyrate (Figure 1B and Figure S1A,C). In the case of other MCFAs, the levels were unaffected or decreased (caproate, caprate (C10), and laurate) but not statistically significantly compared to ad libitum controls (Figure 1B and Figure S1D–G). Importantly, the occurrence of SCFA as well as MCFA in ad libitum-fed and CR mice cecum correlated with the abundance of multiple bacteria (Figure 2). Therefore, to assess the capacity of cecal production of MCFA and considering that SCFAs are products of fibre fermentation by microbiota, the impact of fibre on caprylate production was assessed. Mice were fed custom-made diets high (20%) in soluble fibre (oligofructose from chicory and pectin from apple) or cellulose. A custom-made control diet (Ctrl) with a 5% of fibre mix of cellulose and soluble fibre was used as control. Cellulose had no statistically significant effect compared to control on propionate, butyrate, valerate (C5), caproate, caprylate, caprate, and laurate but it decreased butyrate production (Figure 1C,D and Figure S1H–M). Soluble fibre increased the production of propionate, butyrate, valerate, and caproate (Figure 1D and Figure S1H–M). However, it did not affect the levels of other MCFAs; therefore, other factors than fibre consumption or dietary restriction modulate MCFA production in the gut.

Further, the capacity of utilizing colonic caprylate for energy was verified. For this purpose, mice were rectally infused with ^13^C caprylate and the occurrence of the labelled CO_2_ was measured in breath air. The infusion was followed by a rapid metabolism of caprylate peaking within 15 min and finalized within 1 h (Figure 1E). In order to track if caprylate can be exported from the colon and utilized in other organs, mice were infused with ^13^C caprylate and its distribution was measured in several tissues 1 h following the dosage (Figure 1F). The highest level of ^13^C caprylate was found in the epithelium of the proximal colon, the place of caprylate infusion. Of the tested tissues, caprylate was exported primarily to the liver and the heart but also to WAT, spleen, and skeletal muscle, with the smallest amounts exported to the brain.

**Figure 3:**
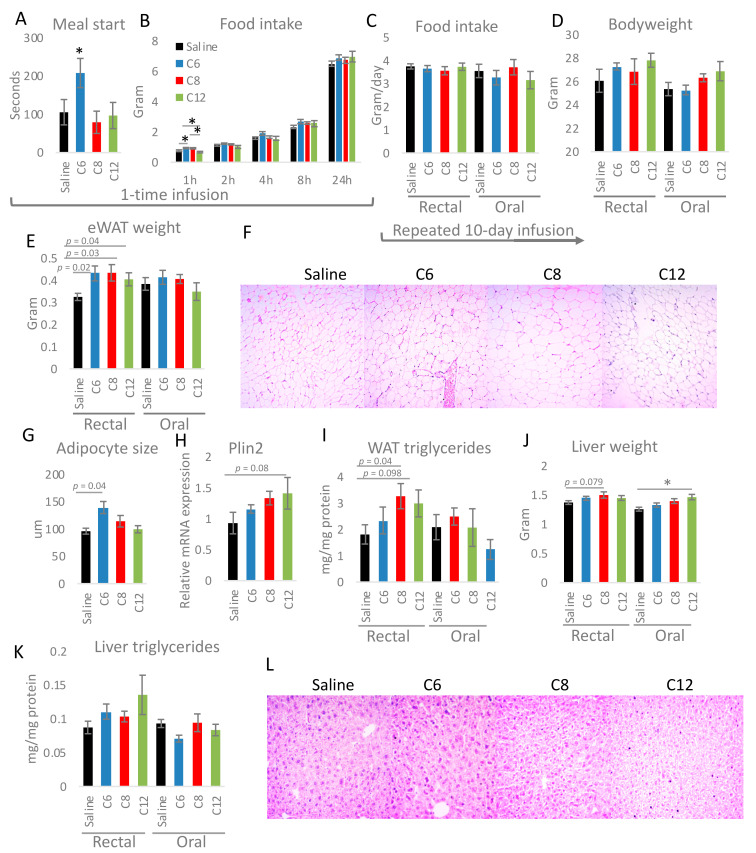
Rectal infusion of MCFA results in changes in epididymal WAT weight, adipocytes size, and lipid accumulation. The speed of meal initiation (**A**) and accumulative food intake over 24 h (**B**) was measured following one-time rectal fatty acid (FA) or saline infusion. Food intake (**C**), body weight (**D**), and epididymal WAT weight (**E**) were compared between animals infused daily rectally or orally with FAs or control solution over 10 days. Adipocyte size was quantified based on hematoxylin & eosin histology staining (**F**,**G**). The mRNA expression of perilipin 2 (Plin2) (**H**) and WAT triglycerides content (**I**) were quantified in mice infused with FA and saline. Liver weight (**J**) and triglycerides content (**K**) were measured for animals infused rectally or orally with FA or control solution. Liver histology was performed to compare lipid accumulation in animals submitted to different treatments (**L**). Statistical significance between the experimental groups was evaluated using ANOVA with Bonferroni correction for multiple testing; *n* = 4–8 for (**G**), *n* = 6–8 for (**I**,**K**), *n* = 8 for other panels; * *p* < 0.05. Data are presented as mean ± SEM.

Next, we assessed if caprylate produced in the cecum or colon and exported to other organs can be utilized to acylate ghrelin and impact appetite. For this, ON fasted mice were rectally infused with 50 μL of 200 μM caprylate or control solutions (caproate, laurate, and saline). Afterwards, the animals’ speed of meal initiation and food intake was measured over 24 h. The rectal infusion of caproate delayed meal initiation; however, caprylate did not affect it (Figure 3A). Food intake following the infusion was higher in caproate and caprylate groups during the first hour of refeeding but then became indistinguishable from the other groups (Figure 3B). Daily infusions of caproate, caprylate, or laurate rectally or orally over 10 days did not change food intake (Figure 3C); however, it resulted in a tendency towards an increase in body weight (Figure 3D) and epididymal WAT (eWAT) in the case of rectal infusion (Figure 3E). Correspondingly, eWAT of mice rectally infused with FAs over 10 days tended to have bigger adipocytes (Figure 3F,G), higher gene expression of lipid droplets-associated protein perilipin (Plin2) (Figure 3H), and more triglycerides (Figure 3I). However, none of the differences were statistically significant. Similarly, the liver of the FA-infused mice had a tendency towards or significantly increased liver weight for rectally and orally dosed FAs, respectively (Figure 3J). Triglyceride content did not differ statistically significantly between the experimental groups (Figure 3K); however, liver histology showed a mild increase in lipid accumulation following FA infusion (Figure 3L). This confirms that MCFAs are exported from the proximal as well as the distal part of the GI tract and utilized in other tissues. To verify if FAs infused over 10 days are completely sequestrated in organs or remain in the circulation we assessed their levels in the blood. The infusion of each FA resulted in a trend towards an increased level of the corresponding FA; however, none were statistically significant (Figure S2A–C).

**Figure 4:**
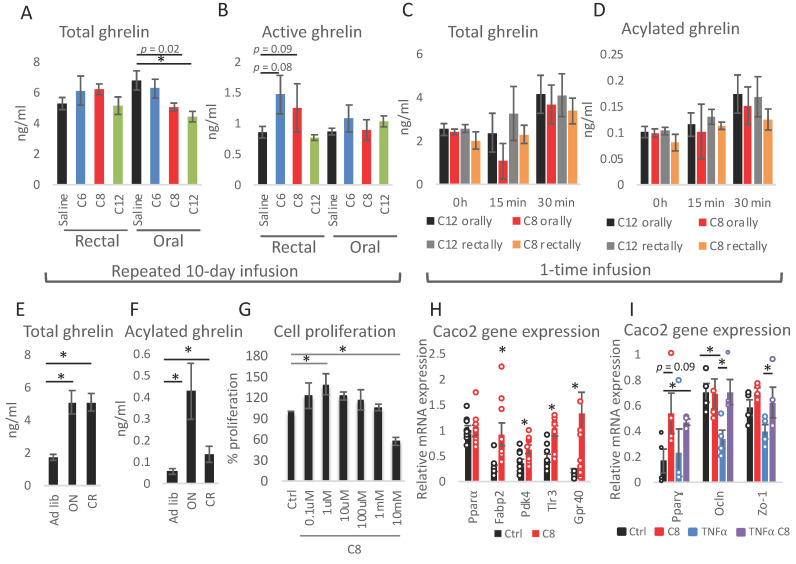
Caprylate affects Caco-2 cell proliferation and gene expression. Total (**A**,**C**,**E**) and active (**B**,**D**,**F**) ghrelin were measured in plasma of mice submitted to 10-day infusion of FAs (**A**,**B**), one-time infusion of caprylate and laurate (**C**,**D**), as well as to ON fasting and CR (**E**,**F**). Caco-2 cell proliferation was measured following control and caprylate treatment for 4 h using a BrdU assay (**G**). Gene expression was measured in Caco-2 cells following caprylate (**H**) or caprylate and TNFα (**I**) treatment. The groups in panels (**A**–**D**,**G**,**I**) were compared using ANOVA with Bonferroni correction for multiple testing. Student’s *t*-test was applied to assess statistical differences between the groups in panels (**E**,**F**) and H; *n* = 8 for (**A**,**F**), *n* = 6 for (**G**), *n* = 9 for (**H**); *n* = 4 for (**I**); * *p* < 0.05. Data are presented as mean ± SEM. Circles in panels H and I indicate values for each biological replicate in the experimental group.

To assess if the FAs exported from the colon can be utilized to acylate ghrelin and to assess the possible difference in the impact of dietary caprylate versus caprylate that may be produced in the colon, ghrelin levels were measured in the plasma of animals from different experimental setups. First, rectal 10-day FAs dosing did not impact plasma total ghrelin while oral infusion decreased ghrelin levels with laurate showing the strongest impact (Figure 4A). Active ghrelin levels were not statistically significantly affected by FAs by any route of dosing; however, rectal C6 and C8 infusion resulted in a trend towards an increased concentration (Figure 4B). To contrast long-term treatments, the immediate impact of FAs was measured. Mice were again infused with caprylate orally or rectally and ghrelin levels were measured in plasma collected before as well as 15 min and 30 min following the infusions. Total (Figure 4C) and acylated ghrelin (Figure 4D) levels were not affected in animals receiving caprylate compared to laurate controls at any of the tested time points. Finally, the impact of the occurrence of colonic caprylate on the levels of total and active ghrelin was verified. The levels of acylated ghrelin were much higher in ON fasted mice than in CR animals (Figure 3E,F), which corresponded to decreased levels of caprylate in CR compared to ON fast mice (Figure 1A,B), indicating a lack of correlation. 

An in vitro Caco-2 cells model was applied to verify the local impact of caprylate on the colon, considering that caprylate may continuously be produced in the gastrointestinal tract but also rapidly metabolized or taken up by the colonocytes. First, the impact of caprylate on in vitro cell proliferation was assessed. Caprylate increased cell proliferation at a concentration of 1 mM and decreased it at 10 mM (Figure 4G). Hence, 1 mM concentration of caprylate was applied for the following experiment. The treatment resulted in an increased expression of fatty acid-binding protein 2 (Fabp2), pyruvate dehydrogenase kinase 4 (Pkd4), toll-like receptor 3 (Tlr3), and Gpr40, but did not affect peroxisome proliferator-activated receptor α (Pparα) (Figure 4H). To assess the impact of caprylate on inflammation, Caco-2 cells were incubated with 10 ng/mL tumour necrosis factor α (TNFα) with and without caprylate (Figure 4I). As previously reported, TNFα resulted in a trend towards reduced zonula occludens-1 (Zo-1) and decreased expression of occludin (Ocln) [68] as well as peroxisome proliferator-activated receptor γ (Pparγ) [69]. Caprylate counterbalanced the impact of TNFα on the expression of these genes (Figure 4I). 

## 4. Discussion

Here we show that caprylate and other MCFAs are present in the mouse cecum. The MCFAs can either be rapidly metabolized in the colon and utilized as a source of energy or be exported to peripheral tissues or stored in the liver and WAT rather than be used to acetylate ghrelin. Further, neither orally nor rectally delivered caprylate serves as a source for ghrelin acylation. Orally and rectally delivered MCFAs show a similar impact on appetite and body weight but rectal caprylate stimulates WAT accumulation. Additionally, locally produced caprylate may impact metabolism, inflammatory parameters, and cell proliferation in the colon.

In previous studies, MCFAs have been delivered with the diet or by injection but until now they have never been studied as endogenous microbiota metabolites. Since MCFAs are taken up rapidly by the intestine it is unlikely that any dietary C8 remains will reach the cecum. Therefore, MCFAs found in the cecum, similar to SCFAs, could result from bacterial fermentation. The consumption of soluble fibre did not stimulate MCFAs production, contrary to its impact on SCFAs which is in line with previous reports [70]. Based on our results, SCFAs are preferentially produced over MCFAs since levels of SCFA were higher than MCFA regardless of experimental conditions. Our efforts to find conditions which enhance MCFAs levels were not successful. However, we can conclude that dietary restriction or increased consumption of soluble and insoluble types of fibre do not increase the production of the majority of MCFAs. This finding is important in the context of dietary restriction of rodents for which hunger leads to increased intake of cage bedding [60] and therefore, to an uncontrolled supplementation with fibre. However, as we presented, cellulose, which is the main component of bedding [60] does not stimulate SCFA and MCFA production.

Typically, the situations in which levels of acylated ghrelin increase are connected with food deprivation. Therefore, it is unlikely that dietary supplementation of caprylate is relevant for its acylation. Rectal MCFA infusion indicated a potentially stimulating impact of these FAs on active ghrelin levels. However, the outcome was mild and was not reflected in the accumulative food intake or body weight changes.

Previous reports showed a strong impact of rectally infused SCFA on FA oxidation and energy expenditure [71] and a difference in the impact of propionate on glucose levels and hepatic cholesterol for rectal versus oral delivery [72]. Similarly, in our study, WAT gain was more pronounced in the mice receiving FAs rectally than those with oral infusion. The difference in the outcomes depending on the delivery route may be the reason why the results of our infusion experiments seem partly contradictory to previous studies reporting that oral MCFAs supplementation reduces body weight, size of adipocytes, and liver weight [16,23,73,74,75]. Notably, the impact on WAT was stronger than on the liver. Further investigation of FA and glucose metabolism in various tissues upon oral and rectal MCFAs infusion is needed to fully understand the impact of the FAs. We conclude that the route of the nutrient delivery may play a crucial role in satiety, metabolism, and FAs trafficking.

Due to the lack of means to block FA uptake, the absolute quantification of SCFAs and MCFAs production in the gut is challenging. During CR, the intestine enhances uptake of certain amino acids and glucose [76]. This may likely be the case also for other nutrients including FAs and it possibly could concern distal parts of the GI tract. It has previously been reported that the colonic absorption of MCFAs exceeds that of SCFAs and that 50–90% of MCFAs are absorbed without being metabolized by the epithelium [77]. However, MCFAs are also an excellent source of energy for colonic enterocytes surpassing even SCFAs [78]. Therefore, it is impossible to state whether the low levels of MCFAs during CR or fasting are due to decreased production, more efficient uptake, or accelerated metabolism. Another factor hindering exact MCFAs quantification is related to the capacity of certain types of fibre to bind and enhance the excretion of lipids [79]. This could further explain why CR-related cage bedding consumption [60] or a high-cellulose diet resulted in the decreased level of some FAs. However, we trust that our approach to measure FAs in cecum instead of faeces contributes to a more accurate picture of FAs production as we assessed the levels at the main place of fibre fermentation and, at the same time, we limited colonic absorption and metabolism.

We showed that caprylate is immediately metabolized, and that it can be exported from the colon to other tissues. Due to this rapid metabolism and transport, the analysis of the impact of the MCFAs on the colon and liver following the infusion is a challenging task. The constant production of MCFAs in the GI tract leads to continuous uptake from the colon but it also results in continuous exposure of the colonic epithelium to the FAs. For that reason, to contrast the sudden bolus of infused FAs and to generate a more real-life resembling situation, we applied an in vitro model and we incubated Caco2 cells with caprylate. The treatment affected genes connected with metabolism, suggesting that the colon may metabolize MCFAs, similarly to SCFAs [78]. Moreover, caprylate increased the expression of GPR40. Importantly, GPR40 stimulates the transcriptional activity of nuclear receptor PPARγ [80], which is an important factor in colon health due to its potent anti-inflammatory and anti-cancerogenic properties [81,82,83,84,85,86]. Noteworthy, MCFAs have been reported to act as PPARγ agonists [87] which corresponds well with the anti-inflammatory properties of MCFAs in the colon [54,56,58]. These results were further confirmed when the role of caprylate was assessed in an inflammatory situation. Caprylate increased the expression of PPARγ in basic conditions as well as counteracted TNFα-triggered reduction in PPARγ expression. Moreover, caprylate modulated expression of the genes coding tight-junction proteins which consequently should impact colon permeability.

In summary, MCFAs produced in the distal GI tract are utilized as a source of energy, have a distinct metabolic faith, but likely do not acylate ghrelin. MCFAs play an anti-inflammatory role in the GI tract. However, it is not possible to deliver oral MCFAs supplements to the distal GI tract due to its rapid uptake via the intestine. Therefore, finding conditions which stimulates MCFAs production may have important therapeutic applications.

## Data Availability

Data is contained within the article or Supplementary Materials.

## Supplementary Materials

The following are available online at https://www.mdpi.com/article/10.3390/nu13113807/s1, Figure S1: The levels of cecal SCFA and MCFA under different dietary conditions, Figure S2: The levels of MCFA in plasma after rectal infusion.
