# Supplementary material for: Colonic Medium-Chain Fatty Acids Act as a Source of Energy and for Colon Maintenance but Are Not Utilized to Acylate Ghrelin
Source: Nutrients. 2021 Oct 26;13(11):3807. doi: 10.3390/nu13113807 (PMC8617845; doi:10.3390/nu13113807)
Supplement: Supplementary file 1 [file nutrients-13-03807-s001.zip › nutrients-1423185-supplementary.pdf]

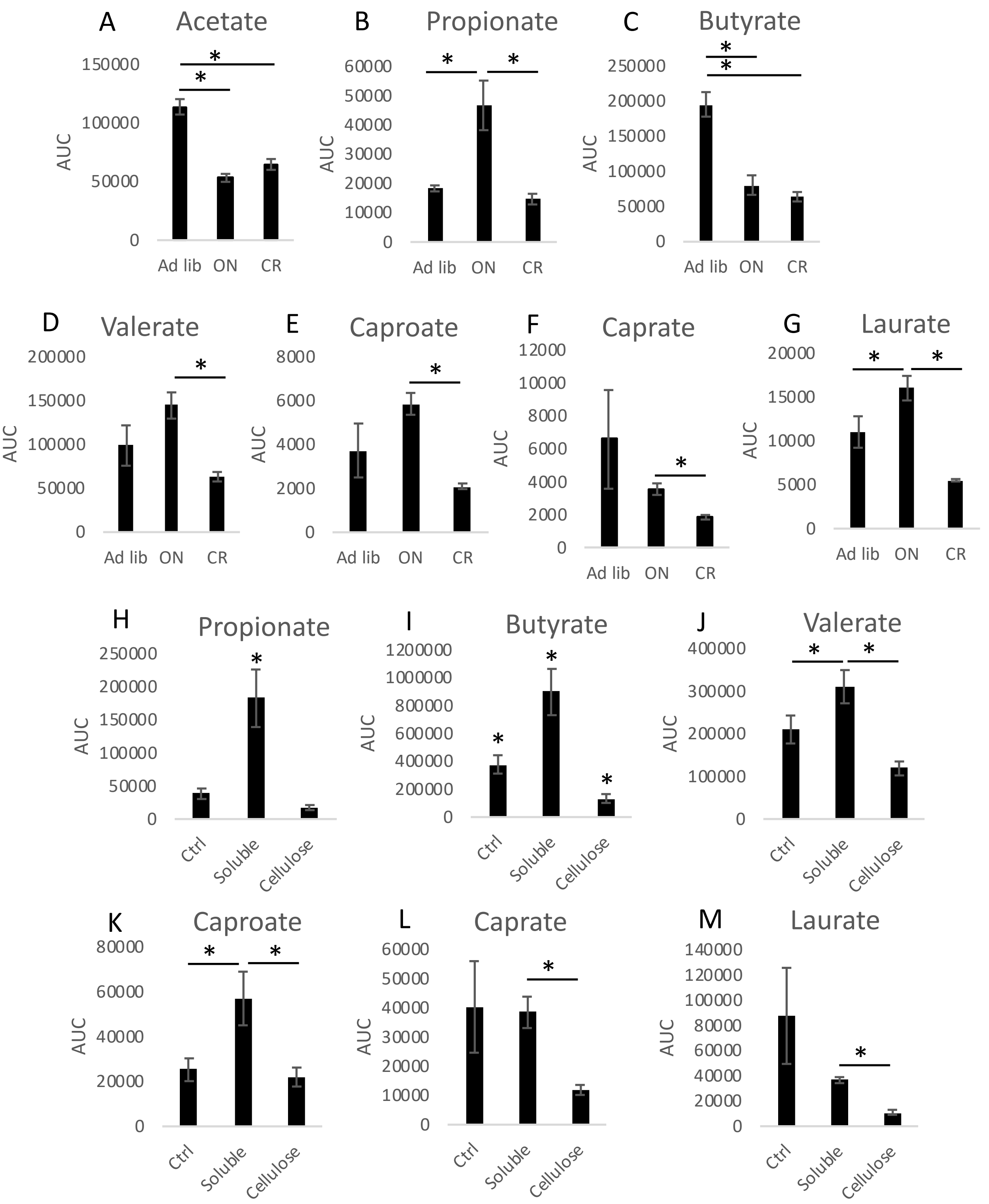

Supplementary Figure S1. The levels of cecal SCFA and MCFA under different dietary conditions. The levels of SCFA and MCFA were measured in the cecum of mice submitted ON fasting or CR (A-G) as well as in mice given high-fiber diets (H-M). The groups were compared using ANOVA with Bonferroni correction for multiple testing. n = 8

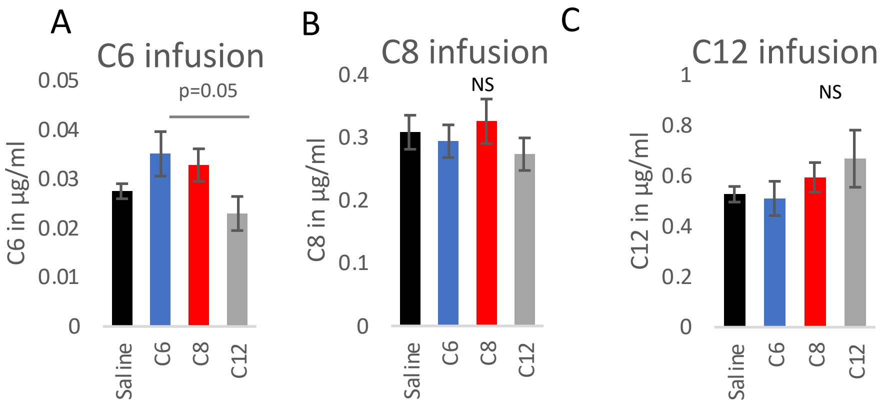

Supplementary Figure S2. The levels of MCFA in plasma after rectal infusion. The levels of caprylate, caproate, and laurate were measured in the plasma of mice infused with the FAs repetitively over 10 days (A). The groups were compared using ANOVA with Bonferroni correction for multiple testing. n = 8

Diet composition

	Control	High-soluble fibre diet	High-cellulose fibre diet
**Ingredient %**			
Casein	21.5	21.5	21.5
Corn starch	35	24	24
Maltodextrin	16	12	12
Sucrose	10	10	10
Cellulose	2.5	20	-
Oligofructose (chicory)	1.25	-	10
Pectin from apple	1.25	-	10
L-Cysteine	0.25	0.25	0.25
Vitamin premix	1	1	1
Mineral premix	6	6	6
Choline chloride	0.2	0.2	0.2
Soybean oil	5	5	5
**Macronutrients**			
Crude protein	19	19	19
Crude fat	5.1	5.1	5.1
Crude fibre	4.8	19.9	19.9
Crude ash	5.4	5.4	5.4
Starch	33.4	23.1	23.1
Sugar	11	11	11.3
**Energy**			
Kcal	15.4	13	13.1
Kcal % protein	21	24	24
Kcal % fat	12	15	15
Kcal % carbohydrates	67	61	61

qPCR primers

Gene name	Forward	Reverse
hFabp2	ATGGCGTTTGACAGCACTTG	TCAGTTCCGTCTGCTAGATTGTA
mFabp2	TGAGCCTGGCATTAGCATGA	AAGGAGTTGAGGCCAAGCGAT
mFas	CAGAAATCGCCTATGGTTGTTG	GCT CAGCTGTGTCTTGGATGC
hGpr40	CTGTACCCCAATCTAGGAGGC	CCAGCGGATTAAGCACCACA
mGpr40	TTTCATAAACCCGGACCTAGGA	CCAGTGACCAGTGGGTTGAGT
Irf7	GAGACTGGCTATTGGGGGAG	GACCGAAATGCTTCCAGGG
mMyD88	GCACCTGTGTCTGGTCCATT	TGTTGGACACCTGGAGACAG
hOcln	TCAGGGAATATCCACCTATCACTTCAG	CATCAGCAGCAGCCATGTACTCTTCAC
mOcln	CCTCCAATGGCAAAGTGAAT	CTCCCCACCTGTCGTGTAGT
P6pc		
hPdk4	CTCCACTGCACCAACGCCTGT	CAAGCCGTAACCAAAACCAGCCAAA
mPepck	AACTGTTGGCTGGCTCTC	GAACCTGGCGTTGAATGC
hPPARα	CTATCATTTGCTGTGGAGATCG	AAGATATCGTCCGGGTGGTT
mPPARα		
hPPARg	GCTGTGCAGGAGATCACAGA	GGGCTCCATAAAGTCACCAA
Pyy	CGGCAGCGGTATGGAAAAAG	GGTCCAAACCTTCTGGCCT
mReg3β	TGGGAATGGAGTAACAATG	GGCAACTTCACCTCACAT
mScd1	GCCCACATGCTCCAAGAGAT	GGGCACTGTCTTCACCTTCT
mStat1	CAGTATGATGAGCACAGTA	AAGTCCTTCAGAGTAACAG
Tlr3		
hZo-1	GGACCAGCTGAAGGACAGCT	TCCGTTAACCATTGCAACTCG
mZo-1	CCACCTCTGTCCAGCTCTTC	CACCGGAGTGATGGTTTTCT
